# Gibberellin Is Involved in Inhibition of Cucumber Growth and Nitrogen Uptake at Suboptimal Root-Zone Temperatures

**DOI:** 10.1371/journal.pone.0156188

**Published:** 2016-05-23

**Authors:** Longqiang Bai, Huihui Deng, Xiaocui Zhang, Xianchang Yu, Yansu Li

**Affiliations:** 1 The Institute of Vegetables and Flowers, Chinese Academy of Agricultural Sciences, Beijing 100081, China; 2 College of Horticulture Science and Engineering, Shandong Agricultural University, Taian 271018, China; Northwest A&F University, CHINA

## Abstract

Suboptimal temperature stress often causes heavy yield losses of vegetables by suppressing plant growth during winter and early spring. Gibberellin acid (GA) has been reported to be involved in plant growth and acquisition of mineral nutrients. However, no studies have evaluated the role of GA in the regulation of growth and nutrient acquisition by vegetables under conditions of suboptimal temperatures in greenhouse. Here, we investigated the roles of GA in the regulation of growth and nitrate acquisition of cucumber (*Cucumis sativus* L.) plants under conditions of short-term suboptimal root-zone temperatures (T_r_). Exposure of cucumber seedlings to a T_r_ of 16°C led to a significant reduction in root growth, and this inhibitory effect was reversed by exogenous application of GA. Expression patterns of several genes encoding key enzymes in GA metabolism were altered by suboptimal T_r_ treatment, and endogenous GA concentrations in cucumber roots were significantly reduced by exposure of cucumber plants to 16°C T_r_, suggesting that inhibition of root growth by suboptimal T_r_ may result from disruption of endogenous GA homeostasis. To further explore the mechanism underlying the GA-dependent cucumber growth under suboptimal T_r_, we studied the effect of suboptimal T_r_ and GA on nitrate uptake, and found that exposure of cucumber seedlings to 16°C T_r_ led to a significant reduction in nitrate uptake rate, and exogenous application GA can alleviate the down-regulation by up regulating the expression of genes associated with nitrate uptake. Finally, we demonstrated that N accumulation in cucumber seedlings under suboptimal T_r_ conditions was improved by exogenous application of GA due probably to both enhanced root growth and nitrate absorption activity. These results indicate that a reduction in endogenous GA concentrations in roots due to down-regulation of GA biosynthesis at transcriptional level may be a key event to underpin the suboptimal T_r_-induced inhibition of root growth and nitrate uptake. These findings may have important practical implications in effective mitigation of suboptimal temperature-induced vegetable loss under greenhouse conditions.

## Introduction

Soil temperatures in greenhouses are often changed slowly and maintained at a suboptimal temperature range for growth of horticultural plants in cold seasons, while air temperature can rise suddenly to high temperatures on sunny days [1]. Therefore, suboptimal root-zone temperature (T_r_) is one of the major limiting factors for winter horticultural production in greenhouse. Xu and Huang [2] suggested that T_r_ is more critical than air temperature in controlling plant growth. Low T_r_ reduces root growth as well as shoots growth even with shoots exposed to optimal temperatures, leading to a heavy loss of crop productivity early in the season when the prices are high [3]. However, the mechanisms underlying the loss of crop productivity by low T_r_ remain largely unknown.

The low T_r_-induced growth suppression has been found to be highly correlated with decrease in nutrient concentrations in plants [4]. Nitrogen (N) is an essential mineral nutrient that often limits plant growth and development. And many studies have shown that N utilization by plants is closely dependent on T_r_. For example, it has been shown that T_r_ can have different effects on N uptake in *Eucalyptus nitens* and rose (*Rosa*×*bybrida cv*. Grand Gala) plants [5, 6]. More recently, Yan et al. found that cucumber (*Cucumis sativus* L.) plants respond more strikingly to N than phosphorus (P) and potassium (K) at low T_r_ [7]. Nitrate assimilation following uptake is the main route by which inorganic N is converted into organic N [8]. However, the capacity to assimilate nitrate by cucumber is not affected by low T_r_, while low T_r_ can severely reduce nitrate absorption [9], suggesting that nitrate uptake is a rate-limiting step for N acquisition under low T_r_ conditions. Given nutrients taken up by plants roots, root growth and physiological activity have important impacts on nutrient absorption. Moreover, it has been shown that both root growth and physiology are inhibited by low T_r_ [3].

Gibberellin acid (GA), a phytohormone produced in roots [4], plays important roles in the regulation of cell expansion and cell proliferation [10–12]. In addition, recent studies also reveal the involvement of GA in regulating plant growth in response to fluctuating environmental conditions [13, 14]. A large body of evidence has shown that ambient temperatures can affect GA signaling pathway. For instance, bioactive GA contents in Arabidopsis were reported to be reduced by cold stress, and accumulation of the nuclear growth-repressing DELLA proteins (DELLAs), key proteins in the GA-signaling pathways, was stimulated, thus leading to a suppression of root growth in Arabidopsis [14]. By contrast, an increase in the ambient temperature stimulates GA production, and reduces DELLAs levels, promoting stem elongation in Arabidopsis [15]. The involvement of GA in temperate-dependent plant growth may imply that GA play a role in the inhibition of plant growth under suboptimal T_r_. However, no study has experimentally tested this hypothesis. Moreover, exogenous application GA_3_ has been reported to promote nitrogen utilization by mustard (*Brassica juncea* L.) [16] and tomato (*Solanum lycopersicum* L.) [17], but few studies have investigated the roles of GA in the regulation of root traits responsible for nutrient absorption.

Cucumber is an important economic crop all over the world. As a chilling-sensitive species, cucumber plants have optimal temperatures for their growth at 24–26/18°C (day/night), with optimal temperatures for root growth at temperatures above 20°C [18]. In this paper, we examined whether GA metabolism is involved in the cucumber growth response to suboptimal T_r_. We further investigated the effect of GA on nitrate uptake at suboptimal T_r_ (16°C). Our results showed that GA played an important role in the regulation of cucumber growth under suboptimal T_r_. Moreover, we found that exogenous application of GA increased the nitrate uptake capacity of cucumber by a negative modulation triggered by reduced concentrations of NO_3_^-^, NH_4_^+^ and the amino acids (Gln and Glu). Finally, our results demonstrated that nitrate uptake of cucumber plants grown under suboptimal T_r_ was improved by modulating GA signaling, thus leading to an increased N accumulation.

## Materials and Methods

### Plant material and growth conditions

Cucumber (*Cucumis sativus* L.), zhongnong 26, was used in this study. Seeds were germinated in darkness at a temperature of 28°C and then grown on vermiculite-sand mixture [1:2, volume/volume (V/V)] with nutrient solutions at 22–25°C /15-18°C (day/night) in the greenhouse with a natural photoperiod [irradiation maxima of around 400 μmol m^−2^ s^−1^ photosynthetic photon flux density (PPFD)]. The nutrient solution (pH 6.0) contained 5 mM KNO_3_, 0.17 mM Ca(H_2_PO_4_)_2_, 1.5 mM CaSO_4_, 0.33 mM MgSO_4_, 25 μM ferric citrate, 3 μM MnSO_4_, 1.7 μM H_3_BO_3_, 0.3 μM CuSO_4_, 0.003 μMZnSO_4_, 0.017 μM Na_2_MoO_4_, 5 mM KNO_3_ [19].

All experiments were conducted in controlled-environment chambers, under a 10-h photoperiod (350 μmol m^-2^ s^−1^) at 25°C during the day and 15°C during the night. Cucumber seedlings were kept at 22°C T_r_ for two additional days prior to the following experiments. In experiments involving growth at two temperatures, seedlings were transferred to 22°C T_r_ or 16°C T_r_ conditions in the presence or absence of exogenous 5 μM GA_3_ for a total of 5 or 8 days. In experiments involving one temperature, 16°C T_r_ only, one group of seedlings was kept at 16°C T_r_ without GA_3_ in the nutrient solution (16°C) and the other groups were transferred to solution with 5 μM GA_3_ (16°C+GA), 5 μM GA_3_ plus 0.5 mM tungstate (W), 5 μM GA_3_ plus 0.25 mM L-methionine sulphoximine (MSX), 5 μM GA_3_ plus 1 mM aminooxyacetate (AOA), or 5 μM GA_3_ plus 0.5 mM azaserine (AZA), which are inhibitors of nitrate reductase, glutamine synthetase, glutamate synthetase and aspartate aminotransferase, respectively [20, 21], for varying periods. Root-zone temperature treatments were achieved by controlling the temperature of nutrient solutions by Low Temperature Thermostat (Safu, Ningbo, China). And the aerial parts of all plants were subjected to the same conditions.

### Growth Parameters

Plant dry weight were measured according to standard methods. Leaves were photographed and area was measured using a LA-S Plant Leaves Analysis software (WSeen, Hangzhou, China). For root morphological parameters, the excised roots were washed in a 1 mM CaSO_4_ solution for 1 min at room temperature before being placed in demineralized water and scanned with the Epson Perfection V850 Pro scan system [Epson(China) Co., Ltd, Shanghai, China]. Root morphological parameters were calculated using a LA-S Plant Roots Analysis software (WSeen, Hangzhou, China).

### N determination

Total nitrogen (Total-N) content was determined using the Kjeldahl method (Hanon K9840 Kjeldahl apparatus), as described by Yan et al. [7].

### GA_4_ determination

The levels of GA_4_ were determined by enzyme-linked immune sorbent assay (ELISA) based on monoclonal antibodies (provided by China Agricultural University, China), as described previously [22].

### Measurement of nitrate influx into roots

Nitrate influx was determined following the protocols described by Garnett et al. [23]. Briefly, on sampling days, plants were transferred to a controlled environment with conditions matching growth conditions (light, temperature and relative humidity) and into solutions identical to growth solutions. The roots were then rinsed thoroughly with the same nutrient solution, followed by 10 min of exposure to the same solution supplemented with ^15^N-labelled NO_3_ (^15^N 20%). At the end of the flux period, roots were rinsed for 2 min in the identical solution, but unlabeled solution. Two identical solutions were used for this rinse to allow an initial 5 s rinse to remove labelled solution adhering to the root surface. Roots were dried at 75°C for 5 d, and then the roots were weighted and ground to a fine powder. Total N and ^15^N in the root samples were determined with an isotope ratio mass spectrometer DELTA plus XP (Thermo Finnigan MAT, Bremen, Germany).

### Quantitative real-time polymerase chain reaction (qPCR)

On sampling days, roots were harvested between 5 and 7 h after the onset of the light period. The whole root was excised and frozen in liquid N_2_ and stored at -80°C. Total RNA from cucumber roots was extracted using RNAprep pure Plant Kit (TANGEN, Beijing, China) according to the manufacturer’s instructions. The concentration of RNA was quantified by spectrophotomatrical measurement at λ = 260 nm, and its integrity was checked on agarose gels. First strand cDNA was synthesize using FastQuant RT Kit (TANGEN, Beijing, China) according to the manufacturer’s instructions, and used as templates in the Amplification assay. qPCR and melting curve analysis were performed following the manufacturer’s instructions of the SuperReal PreMix Plus (SYBR Green) Kit (TANGEN, Beijing, China) on the Applied BioSystems 7500 Real Time PCR System (Applied BioSystems) with specific primers (S1 Table).The reaction mixture had a final volume of 20 μl, containing 10 μl 2×SYBR Premix Ex TaqTMII, 0.4 μl of each primer, 0.4 μl 50 × ROX Reference Dye II and 8.4 μl of 5-fold dilution cDNA-template. The thermal cycling conditions were as follows: 95°C for 15 min (1 cycle), 95°C for 10 s, 60°C for 20 s and 72°C for 32 s (40 cycles) and 72°C for 5 min (1 cycle). For each qPCR experiment, no cDNA-template controls were performed to ensure that reagents and RNA samples were free of genomic DNA contamination. The amplifications were performed on three independent samples for each treatment and triplicate reactions were carried out for each sample, in 96-well plates. For relative quantification, *Actin* was detected as an internal reference, and the 2^−ΔΔCt^ method was used. The primers were designed very carefully to ensure amplification of single gene isoforms using the Primer Premier 5 software [24]. To confirm the specificity of amplification, melting curve analysis was performed using the method as recommended by the manufacturer of Stratagene Mx3000p system to identify putative unspecific PCR products.

### Statistical analyses

Statistical analyses were performed with the Data Processing System (DPS) version 7.05 software [25]. Data are presented as mean values±SE. Differences between treatments were analyzed by Least-Significant Difference (LSD), taking *P<0*.*05* as a significant difference.

## Results

### Effects of suboptimal T_r_ and exogenous GA application on the growth of cucumber seedlings

**Fig 1:**
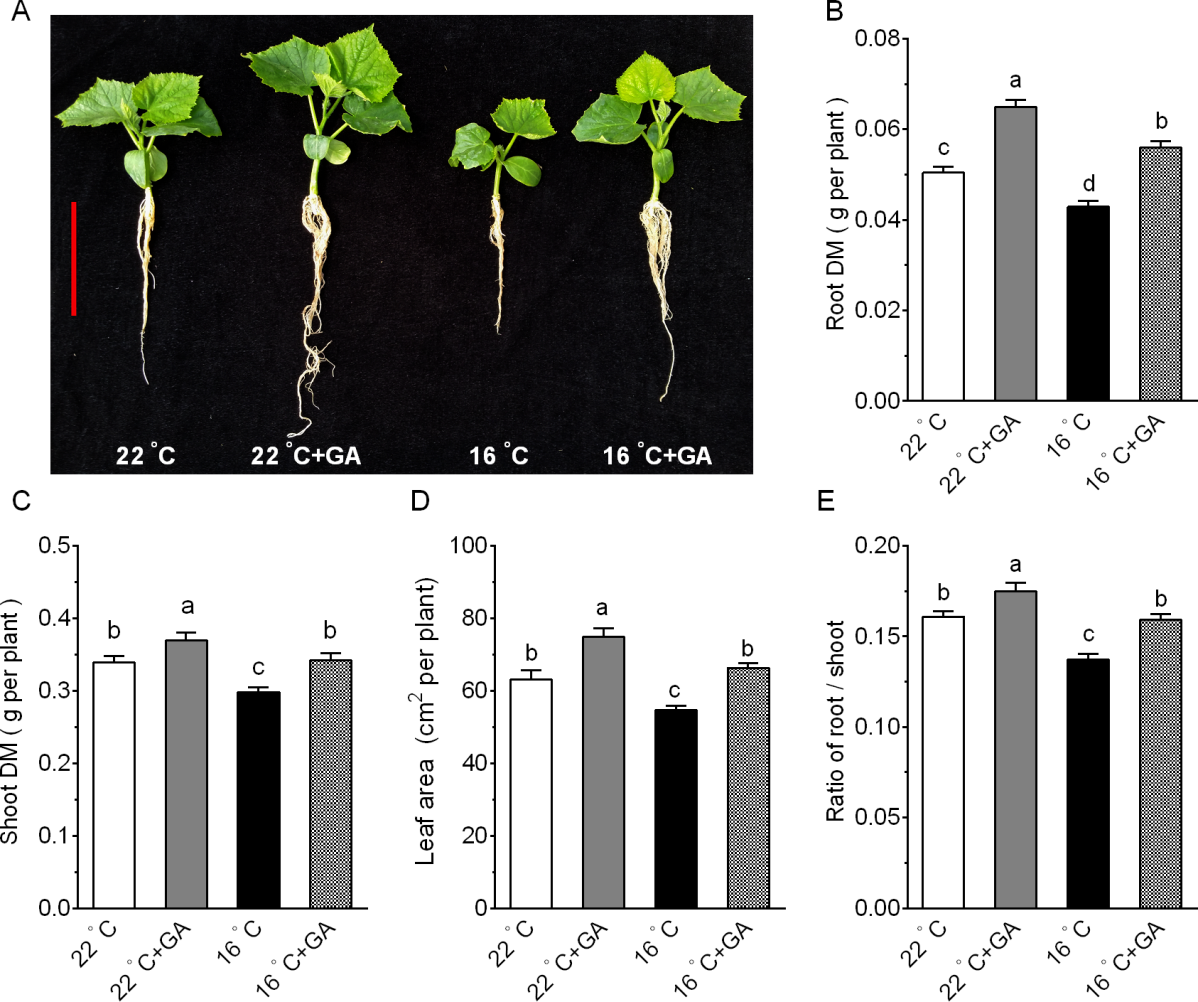
Effect of suboptimal T_r_ and GA on the growth of cucumber seedlings. (A) Phenotypes of cucumber seedlings. (B) Root dry mass (DM) of cucumber seedlings. (C) Shoot DM of cucumber seedlings. (D) Leaf area of cucumber seedlings. (E) Root to shoot ratio of cucumber seedlings. 15-day-old cucumber seedlings were transferred to 22°C T_r_ and 16°C T_r_ conditions in the presence or absence of GA 5 μM GA for 8d. Data are means±SE. Different letters on the top of column indicate significant differences (*P <0*.*05*, n = 6). Bar = 10 cm.

**Fig 2:**
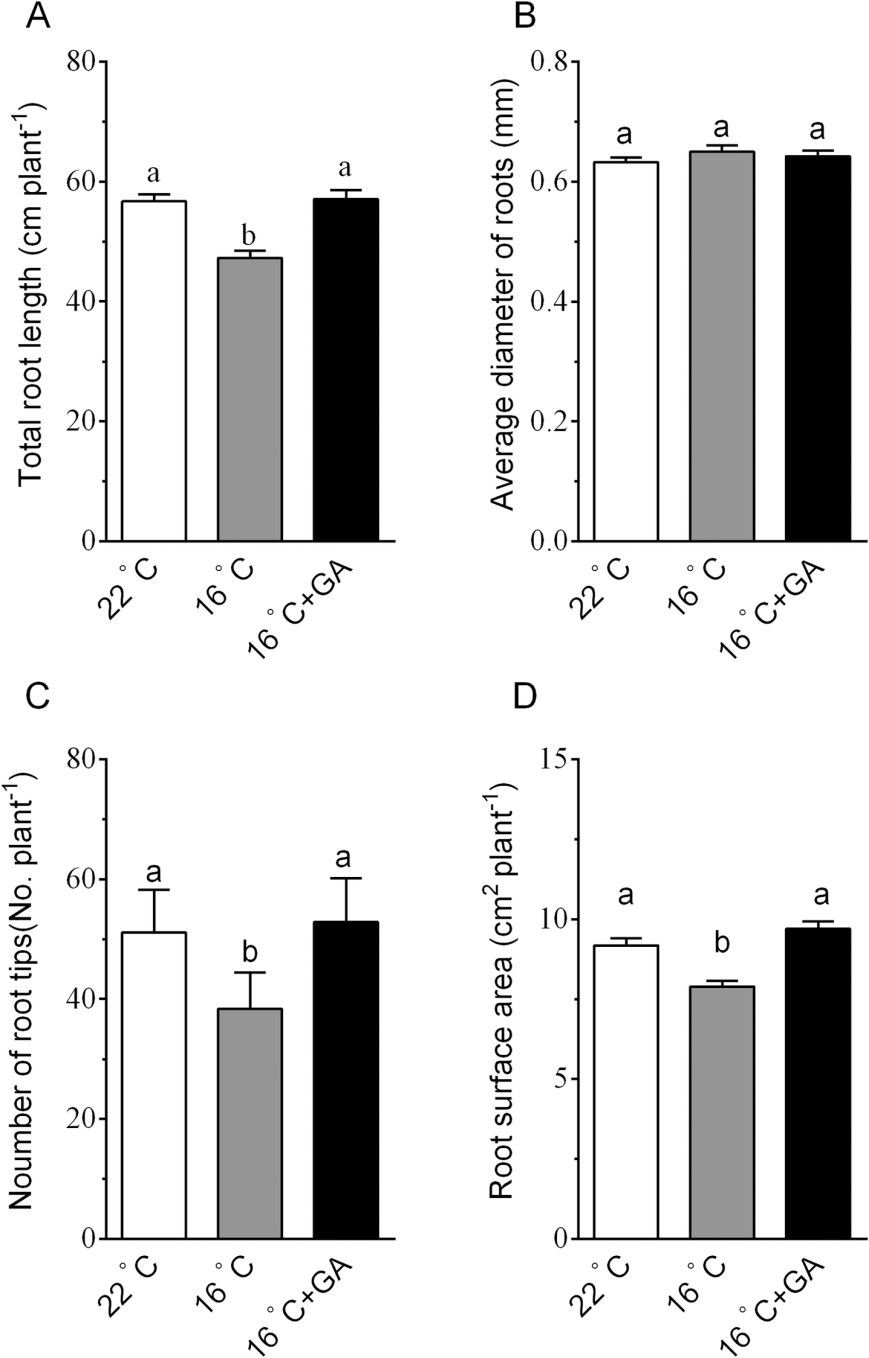
Effects of suboptimal T_r_ and GA on root morphological parameters of cucumber seedlings. (A) Total root length of cucumber seedlings. (B) Average diameter of roots of cucumber seedlings. (C) Number of root tips of cucumber seedlings. (D) Root surface area of cucumber seedlings. 10-day-old seedlings were transferred to 16°C T_r_ conditions in the presence or absence of exogenous 5 μM GA for 5 d. Data are means±se. Different letters on the top of column indicate significant differences (*P <0*.*05*, n = 6).

Previous studies showed that suboptimal root-zone temperate (T_r_) suppressed plant growth and productivity by inhibiting root growth and function [3]. To test whether GA is involved in the suboptimal T_r_-induced suppression of cucumber growth, we compared growth of cucumber grown at 22°C T_r_ and 16°C T_r_ in the presence or absence of GA. Cucumber seedlings exposed to 16°C T_r_ for 8 days exhibited visibly growth retardation compared to those seedlings grown at 22°C T_r_ (Fig 1A). And this was confirmed by quantitative analysis. After 8 days of 16°C T_r_ treatment, shoot dry weigh, root dry weight, and leaf area of cucumber seedlings significantly decreased by 12.1%, 14.9% and 13.3%, respectively (Fig 1B–1D). However, exogenous GA restored the growth of cucumber grown at suboptimal T_r._ GA increased shoot dry weigh, root dry weight, and leaf area of cucumber grown at 16°C T_r_ conditions by 14.8%, 30.5% and 22.6%, respectively. And similar results were obtained using 20-day-old seedlings as indicated in S1 Fig. Meanwhile, shoot dry weigh, root dry weight, and leaf area of cucumber grown at 22°C T_r_ in the presence of GA increased by 8.9%, 28.8% and 16.2%, respectively, compared with those at 22°C T_r_ in the absence of GA. Root/shoot ratio at 16°C T_r_ was significantly lower than at control of 22°C T_r_, and the reduced ratio was reversed by exogenous application of GA (Fig 1E). Root morphological parameters, such as total root length, root tip number and root surface area, were significantly reduced by suboptimal T_r_ treatment, and GA application reversed the reduction (Fig 2A, 2C and 2D). Average diameter of cucumber roots were not affected by suboptimal T_r_ or GA treatment (Fig 2B). These results suggest that the reduction in growth by suboptimal T_r_ may result from a reduction in endogenous GA level in roots.

**Fig 3:**
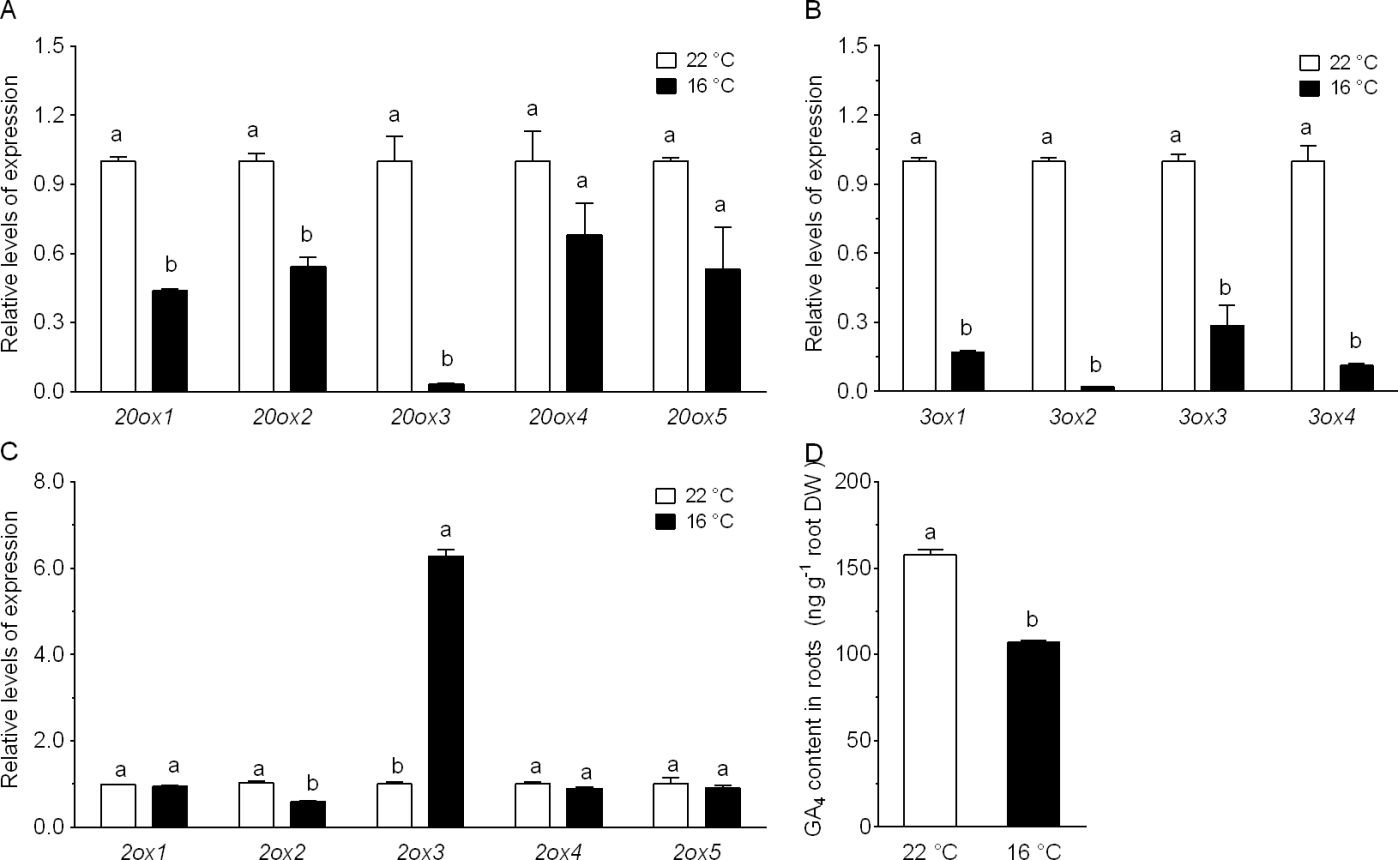
Suboptimal T_r_ regulates the transcript levels of GA biosynthesis genes. (A) Expression profiles of GA biosynthesis *GA 20-oxidase* genes. (B) Expression profiles of *GA 3-oxidase* genes. (C) Expression profiles of *GA 2-oxidase* genes. (D) Determination of GA_4_ concentration in cucumber roots. 10-day-old seedlings were treated with 22°C T_r_ or 16°C T_r_ for 5 d. Data are means±SE. Different letters on the top of column indicate significant differences (*P <0*.*05*, n = 3).

Bioactive GA concentration in plants is tightly regulated at transcriptional level by changes in the expression of those genes encoding enzymes for biosynthesis and deactivation of bioactive GA, including GA 20-oxidaxes (GA20ox) and GA 3-oxidases (GA3ox), and GA 2-oxidases (GA2ox) [26]. To test whether the reduced root growth by suboptimal T_r_ results from altered GA metabolism, we analyzed the effects of suboptimal T_r_ on transcript levels of *CsGA20ox*, *CsGA3ox*, and *CsGA2ox* by qPCR. As shown in Fig 3, expression of genes belonging to *GA20ox* family (*CsGA20ox1-3*) and *GA3ox* family (*CsGA3ox1*-*4*) was significantly reduced upon exposure to suboptimal temperate at 16°C, while the transcript of *CsGA20ox4* and *CsGA20ox5* remained relatively unchanged by the same suboptimal T_r_ treatment. In contrast, there was a marked up-regulation of *CsGA2ox3* expression after transferring seedlings from 22°C T_r_ to 16°C T_r_, while a significant down-regulation of *CsGA2ox2* was observed by the low T_r_ treatment (Fig 3C).

The observed changes in expression of genes responsible for GA homeostasis prompt us to examine whether exposure of cucumber seedlings to suboptimal T_r_ alter endogenous GA concentrations. Our results showed that GA_4_ concentration in roots of cucumber grown at 16°C T_r_ was significantly less than that in seedlings grown at 22°C T_r_ (Fig 3D). These results suggest that modulation in GA metabolism is recruited by cucumber to re-program its growth in response to suboptimal T_r_.

### Effects of suboptimal T_r_ and exogenous GA application on ^15^NO_3_^-^ influx

**Fig 4:**
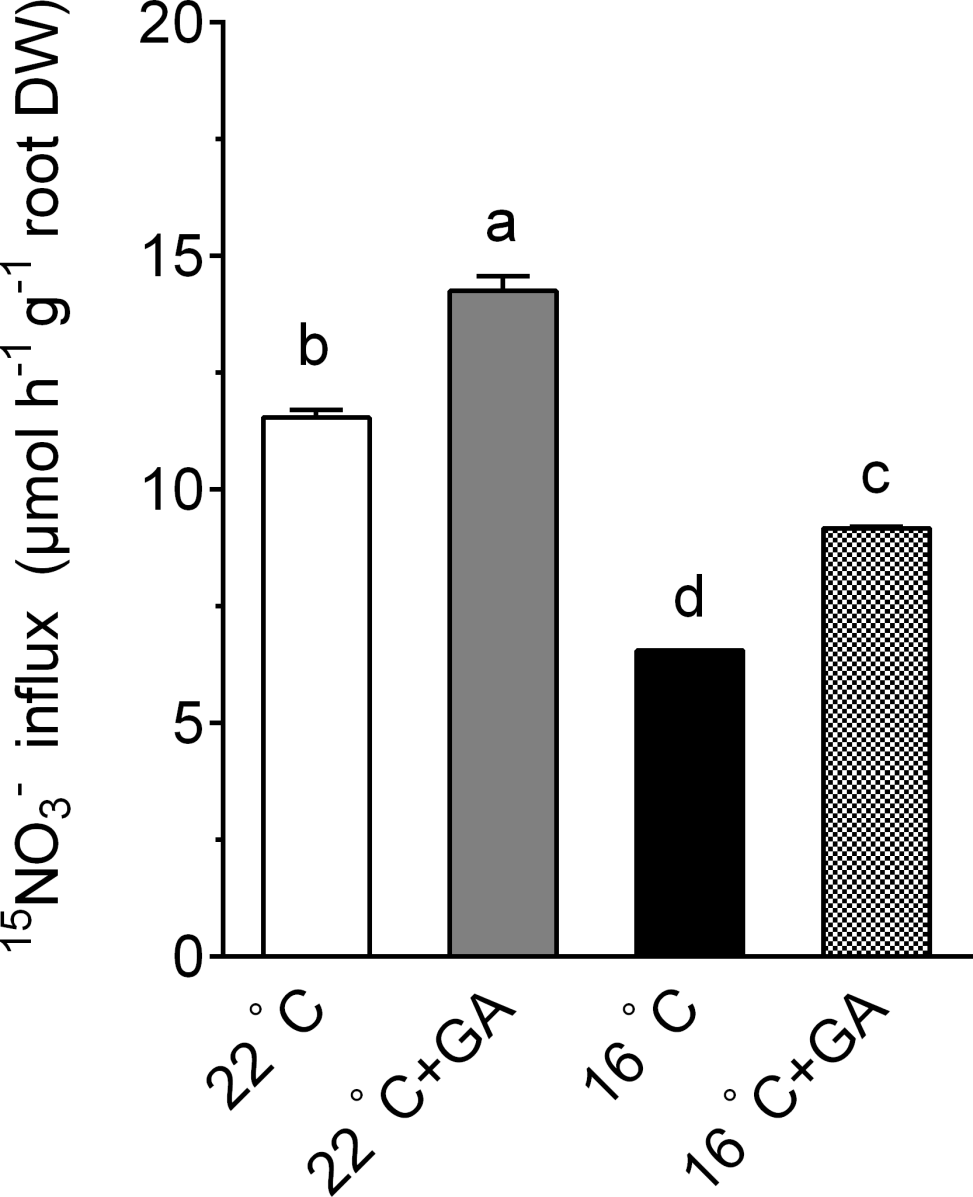
Effect of suboptimal T_r_ and GA on ^15^NO_3_^-^ influx of cucumber. 15-day-old cucumber seedlings were transferred to 22°C T_r_ and 16°C T_r_ conditions in the presence or absence of GA 5 μM GA for 8d. Data are means±SE. Different letters on the top of column indicate significant differences (*P <0*.*05*, n = 3).

It was proposed that reduced N uptake at low T_r_ play an important role in modulation of plant growth in response to low T_r_ [27]. To explore the mechanism underlying the GA-dependent cucumber growth under suboptimal T_r_, we investigated the effect of suboptimal T_r_ and GA on nitrate uptake of cucumber. As shown in Fig 4, ^15^NO_3_^-^ influx into roots of seedlings grown at 16°C T_r_ was decreased by 43.2% compared to that of seedlings grown at 22°C T_r_, whereas exogenous GA increased ^15^NO_3_^-^ influx of cucumber grown at 16°C T_r_ conditions by 39.9%. Meanwhile, GA also increased ^15^NO_3_^-^ influx of cucumber grown at 22°C T_r_ conditions by 23.6%, a degree lower than that in 16°C T_r_ conditions.

### Effects of suboptimal T_r_ and exogenous GA application on *CsNRT1* expression

**Fig 5:**
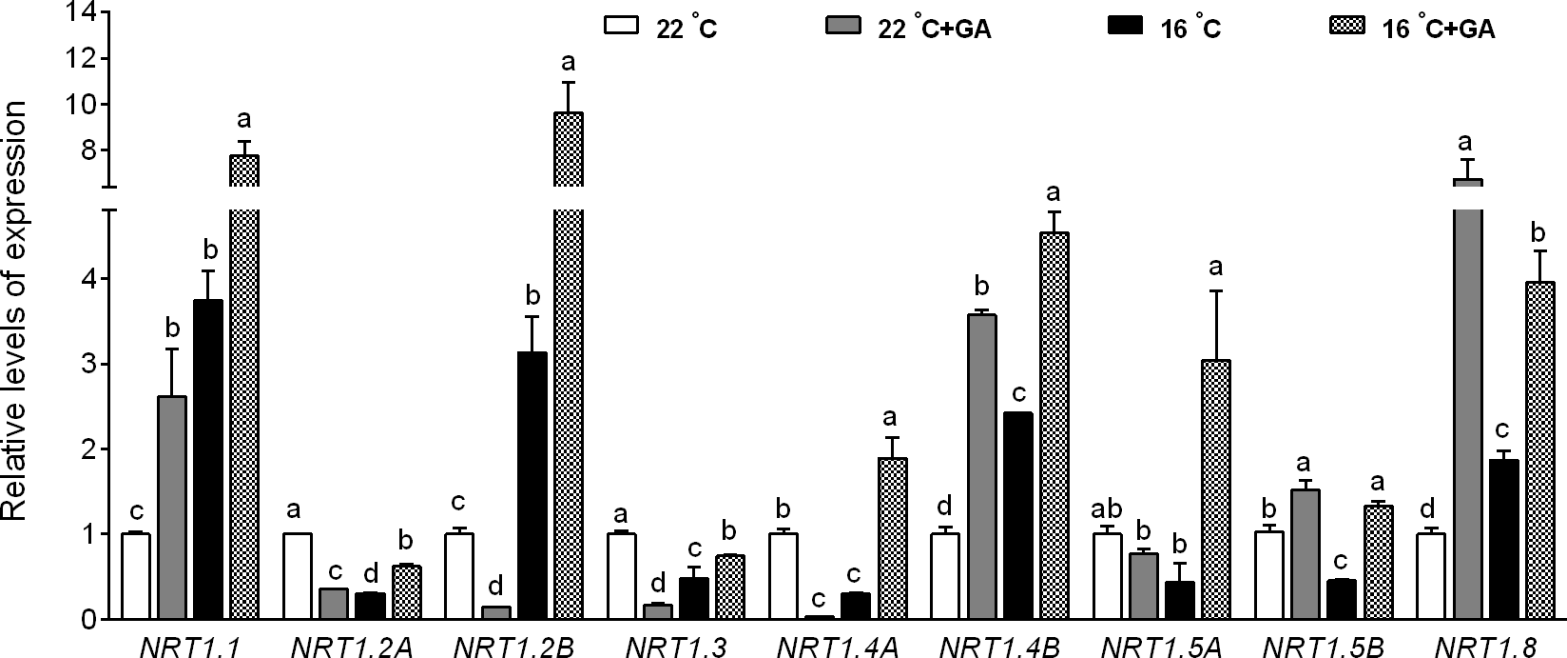
Suboptimal T_r_ and GA regulate the transcript levels of *CsNRT1* family genes. 15-day-old cucumber seedlings were transferred to 22°C T_r_ and 16°C T_r_ conditions in the presence or absence of GA 5 μM GA for 8 d. The relative expression levels were analyzed by qPCR using *Actin* as internal control. Data are means±SE. Different letters on the top of column indicate significant differences (*P <0*.*05*, n = 3).

Many studies have shown that regulation of nitrate uptake is often highly correlated with changes in expression of *NRT* genes [28]. We next analyzed the effects of suboptimal T_r_ and exogenous GA on the transcript of *CsNRT1* which encodes low affinity nitrate transporter system (LATS) nitrate transporters [19]. Exposure of cucumber seedlings to 16°C T_r_ decreased the transcription levels of *CsNRT1*.*2A*, *CsNRT1*.*3*, *CsNRT1*.*4A*, *CsNRT1*.*5A* and *CsNRT1*.*5B*, whereas significantly enhanced the transcription levels of *CsNRT1*.*1*, *CsNRT1*.*2B*, *CsNRT1*.*4B* and *CsNRT1*.*8*. GA application at 16°C T_r_ led to a significant up-regulation of all the 9 *CsNRT1* genes expression. By contrast, GA application at 22°C T_r_ only increased the transcription levels of *CsNRT1*.*1*, *CsNRT1*.*4B*, *CsNRT1*.*5B* and *CsNRT1*.*8* (Fig 5).

### Effects of inhibitors of N assimilation on ^15^NO_3_^-^ influx of cucumber grown at 16°C T_r_ with GA

**Fig 6:**
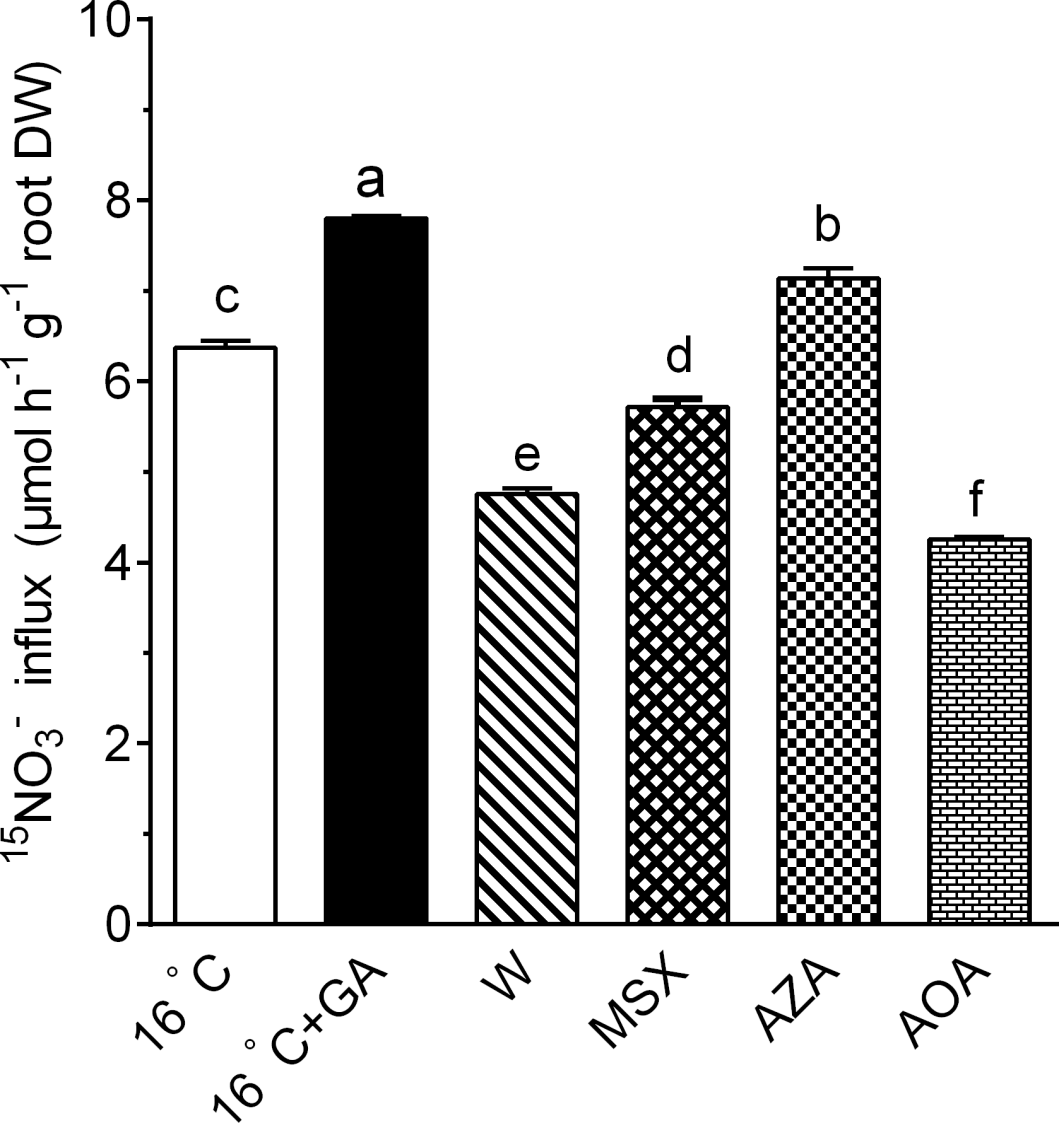
Effects of inhibitors of key enzymes in N assimilation on ^15^NO_3_^-^ influx of cucumber seedlings. 20-day-old cucumber seedlings were exposed for 6 h to 16°C T_r_ (16°C), 16°C T_r_ in the presence of 5 μM GA (16°C+GA), 5 μM GA plus 0.5 mM tungstate (W), 5 μM GA plus 0.25 mM L-methionine sulphoximine (MSX), 5 μM GA plus 0.5 mM azaserine (AZA), and 5 μM GA plus 1 mM aminooxyacetate (AOA). Data are means±SE. Different letters on the top of column indicate significant differences (*P <0*.*05*, n = 3).

It has long been recognized that root nitrate uptake rate is regulated by feedback repression of N-metabolites [8, 29]. To investigate whether the modulation of root NO_3_^-^ uptake by GA treatment at 16°C T_r_ is associated with the feedback mechanism, the inhibitors of key enzymes of the N assimilation were used to increase tissue concentrations of NO_3_^-^, NH_4_^+^, Gln and Glu [20, 21], and their effects on ^15^NO_3_^-^ uptake by cucumber roots were examined. Compared with the GA treatment at 16 C T_r_, application of tungstate, MSX and AZA significantly reduced the ^15^NO_3_^-^ influx by 39.0%, 26.7% and 45.5%, while treatment with AOA only reduced ^15^NO_3_^-^ influx by 8.6% (Fig 6).

### Effects of suboptimal T_r_ and exogenous GA application on tissue N concentration and total N accumulation of cucumber seedlings

**Fig 7:**
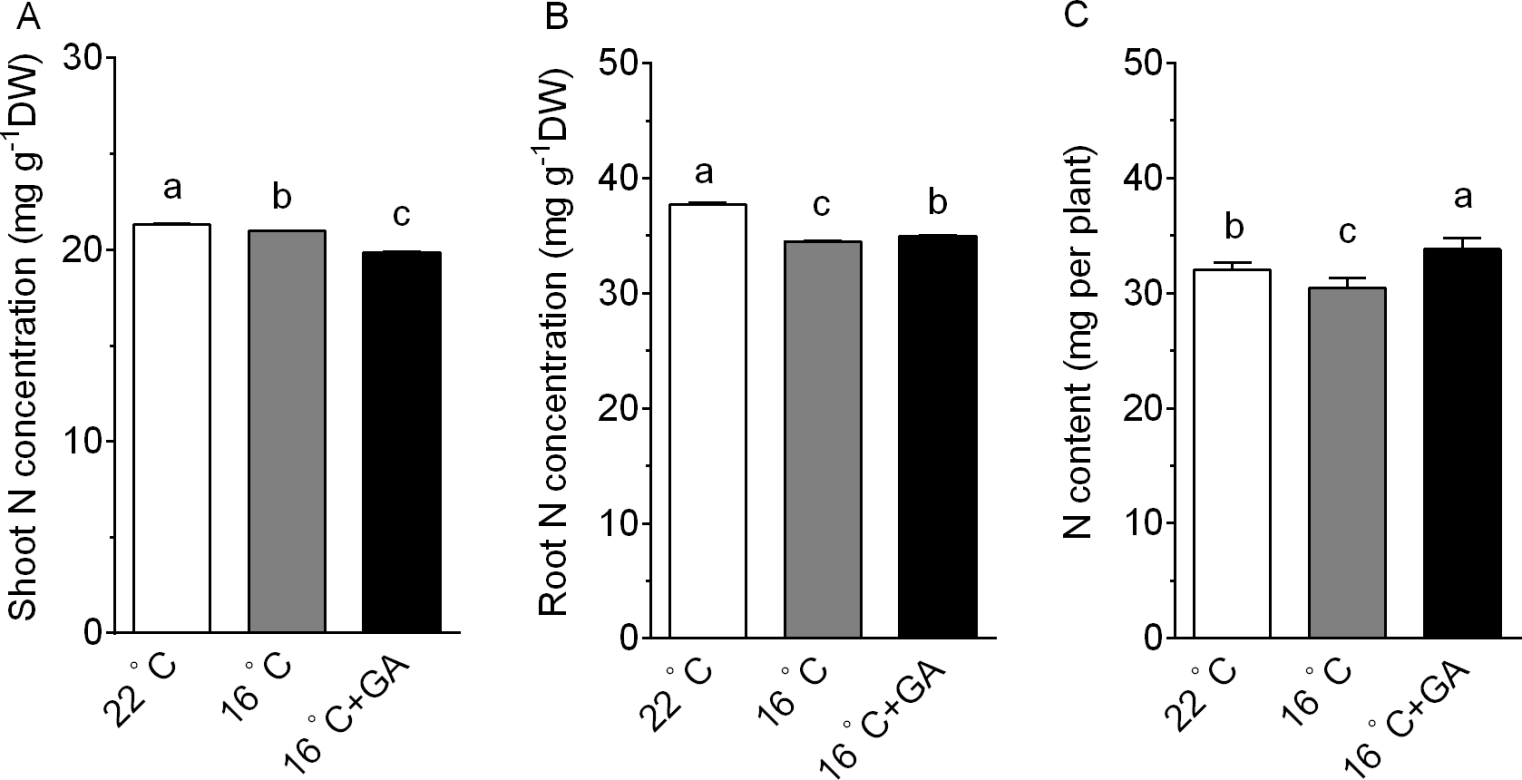
Comparison of N concentration in shoots (A), N concentration in roots (B) and N accumulation (C) of cucumber seedlings. 20-day-old cucumber seedlings were transferred to 22°C T_r_, and 16°C T_r_ conditions with or without 5 μM GA for 8 d. Data are means±SE. Different letters on the top of column indicate significant differences (*P <0*.*05*, n = 6).

As shown in Fig 7A, N concentration in shoots of seedlings grown at 16°C T_r_ without GA was decreased by 1.5% compared to that of seedlings grown at 22°C T_r_. However, seedlings grown at 16 C T_r_ with GA had a much lower shoot N concentration. N concentration in roots was also decreased by 16°C T_r_ treatment. Whereas exogenous GA increased N concentration in roots of cucumber grown at 16°C T_r_ by 1.4% (Fig 7B).

Exposure of cucumber seedlings to 16°C T_r_ resulted in a significant decrease in N accumulation (Fig 7C). However, due to GA-promoted increases in shoot DM (S1 Fig), exogenous GA led to a significant increase in N accumulation of whole plants by 11.1% (Fig 6C), which were even higher than those at 22°C T_r_ plants (Fig 7C).

## Discussion

Under low T_r_, root growth and root morphology as well as shoot growth and leaf area expansion are negatively affected [4]. Our findings that exposure of cucumber seedlings to 16°C T_r_ resulted in a suppression of root and shoot growth are in agreement with this notion. Moreover, we demonstrated that the suboptimal T_r_-induced inhibition of growth in cucumber plants was reversed by exogenous application of GA (Fig 1). This finding may imply that GA is involved in the response of cucumber to suboptimal T_r_. We further monitored the responses of genes encoding GA metabolic enzymes to suboptimal T_r_ treatment at the transcriptional level. Our results revealed that suboptimal T_r_ decreased the levels of *CsGA20ox2*, *CsGA20ox3* and *CsGA3ox2* transcripts that encode enzymes to synthesize bioactive GAs (Fig 3A and 3B), and increased the level of *CsGA2ox3* transcripts encoding an enzyme that deactivates bioactive GA (Fig 3C). The suppressed GA biosynthesis and enhanced GA deactivation at the transcriptional level may account for the observed reduction in GA_4_ concentrations in roots under suboptimal T_r_ (Fig 3D). Our findings are in line with those findings in stem tissues of pea (*Pisum sativum*) plants exposed to suboptimal air temperature [30]. And the down-regulation of *CsGA2ox2* may result from the feed-forward regulatory mechanisms underlying the GA homeostasis [30]. GA application at optimal T_r_ also promoted the growth of cucumber seedlings, in which the response to GA was smaller than in plants grown at suboptimal T_r_ conditions. This may caused by exogenous GA application caused excess GA in tissue, since bioactive GA in seedlings grown in optimal T_r_ at the normal level, and activated the feedback regulation mechanism to maintain GA homeostasis [30].

In addition to suppression of growth, we also showed that exposure of cucumber plants to suboptimal T_r_ caused a significant reduction in nitrate uptake into roots (Fig 4). This observation agrees with the reports that low T_r_ can inhibit nutrient uptake by plants [4, 7]. One important finding in the present study is that exogenous application of GA to suboptimal T_r_-treated cucumber plants can markedly mitigate the suboptimal T_r_-induced inhibition of nitrate uptake (Fig 4).

Studies suggest that different isoforms of *NRT* genes play specific roles in nitrate absorption [31]. Thus, expression profiles of the *CsNRT1* genes were further analyzed in the roots. All of the 9 *CsNRT1* genes were dramatically induced by GA application under suboptimal T_r_ condition, meanwhile, 4 of the 9 genes were induced by GA application under optimal T_r_ condition (Fig 5), suggesting that GA regulates cucumber nitrate absorption at transcription level. However, *CsNRT1*.*1*, *CsNRT1*.*2B*, *CsNRT1*.*4B* and *CsNRT1*.*8* showed increased expression levels in seedling exposed to suboptimal T_r_ for 8 days (Fig 5), while nitrate uptake rate was suppressed by the same GA treatment (Fig 4). The hypothesis that different members of the same gene family may demonstrate differential expression to balance the gene expression and metabolic product profiles [32], may account for these results.

It has been well established that N acquisition in plants is regulated by phytohormones in general [33, 34], and CK in particular, which may act as a status signal of nitrogen to inhibit nitrate uptake in root [34, 35]. Khan et al. [16] reported that exogenous application of GA_3_ enhanced N uptake and stimulated shoot growth in mustard (*Brassica juncea* L.), but the underlying mechanism is not known yet. It has been suggested that nitrate influx is under a negative control of tissue N-metabolites content, which is associated with N demand for plant growth [36]. Thus, we speculated that GA may regulate nitrate acquisition capacity of cucumber plants by affecting concentrations of N assimilation products (NO_3_^-^ and/or products of its assimilation). And our results revealed that GA-induced increase in ^15^NO_3_^-^ influx into cucumber roots was reduced by inhibitors of enzymes responsible for N assimilation (Fig 6). This observation provides strong evidence supporting that GA enhanced nitrate acquisition capacity of cucumber plants by a negative feedback signal associated with reduced concentrations of NO_3_^-^, NH_4_^+^ and the amino acids Gln and Glu due to enhanced growth.

Environmental stresses such as low and high temperatures, drought and salinity frequently limit crop growth and yield. These abiotic stresses can result in more than 50% loss of crop yield worldwide every year [37]. An increase in tolerance of crops to stresses could improve yield stability. Over recent years, most studies have focused on generating transgenic plants by expressing stress-related genes, such as *DREB*/*CBF* genes, to combat the environmental stresses. Unfortunately, the transgenic plants often suffer from stunted growth and reduced yield potential even under optimal conditions [38, 39]. Furthermore, these extreme stress situations hardly occur in modern agricultural practices. Most commonly encountered abiotic stress by crops may be mild stress. Therefore, a more promising strategy to reduce the growth sensitivity to moderate stress is needed, such that accumulation of a maximal biomass during their life cycle ultimately leads to a high yield [37]. To date, extensive effort has been made to elucidate the mechanisms underlying the stress-induced inhibition of growth. Although plants have evolved several pathways to cope with the adverse environments [40, 41], GA-dependent growth regulation appears to be conserved across different stresses, and may be a convergence point for other pathways [37, 42]. Here, we showed that cucumber growth was markedly suppressed by suboptimal T_r_, and that this inhibition was reversed by exogenous application of GA (Fig 1 and S1 Fig). We further demonstrated that application of GA can improve cucumber seedlings biomass and N accumulation (S1 Fig and Fig 7) under suboptimal T_r_ conditions. These findings also provide valuable clues to further investigate the roles of GA in modulation of vegetable growth and N use efficiency under suboptimal growth conditions.

## Conclusions

Suboptimal root-zone temperature restrains cucumber seedling growth partly by reducing the level of bioactive GA. And the reduction in bioactive GA concentration modulates the root nitrate uptake via the signaling cascades associated with N status in cucumber plants.

## Supporting Information

S1 Fig Effect of suboptimal T_r_ and GA on the growth of cucumber seedlings. (A) phenotypes of cucumber seedlings. (B) root DM of cucumber seedlings. (C) shoot DM of cucumber seedlings. (D) leaf area of cucumber seedlings. E, root to shoot ratio of cucumber seedlings. 20-day-old cucumber seedlings were transferred to 22 °C T_r_ and 16 °C T_r_ conditions in the presence or absence of GA 5 μM GA for 8d. Data are means±SE. Different letters on the top of column indicate significant differences (*P <0*.*05*, n = 6).
(TIF)

S1 Table The list of gene specific primes for qPCR. (DOCX)
