# Supplementary figures and images for: Gibberellin Is Involved in Inhibition of Cucumber Growth and Nitrogen Uptake at Suboptimal Root-Zone Temperatures
Source: PLoS One. 2016 May 23;11(5):e0156188. doi: 10.1371/journal.pone.0156188 (PMC4877016; doi:10.1371/journal.pone.0156188)

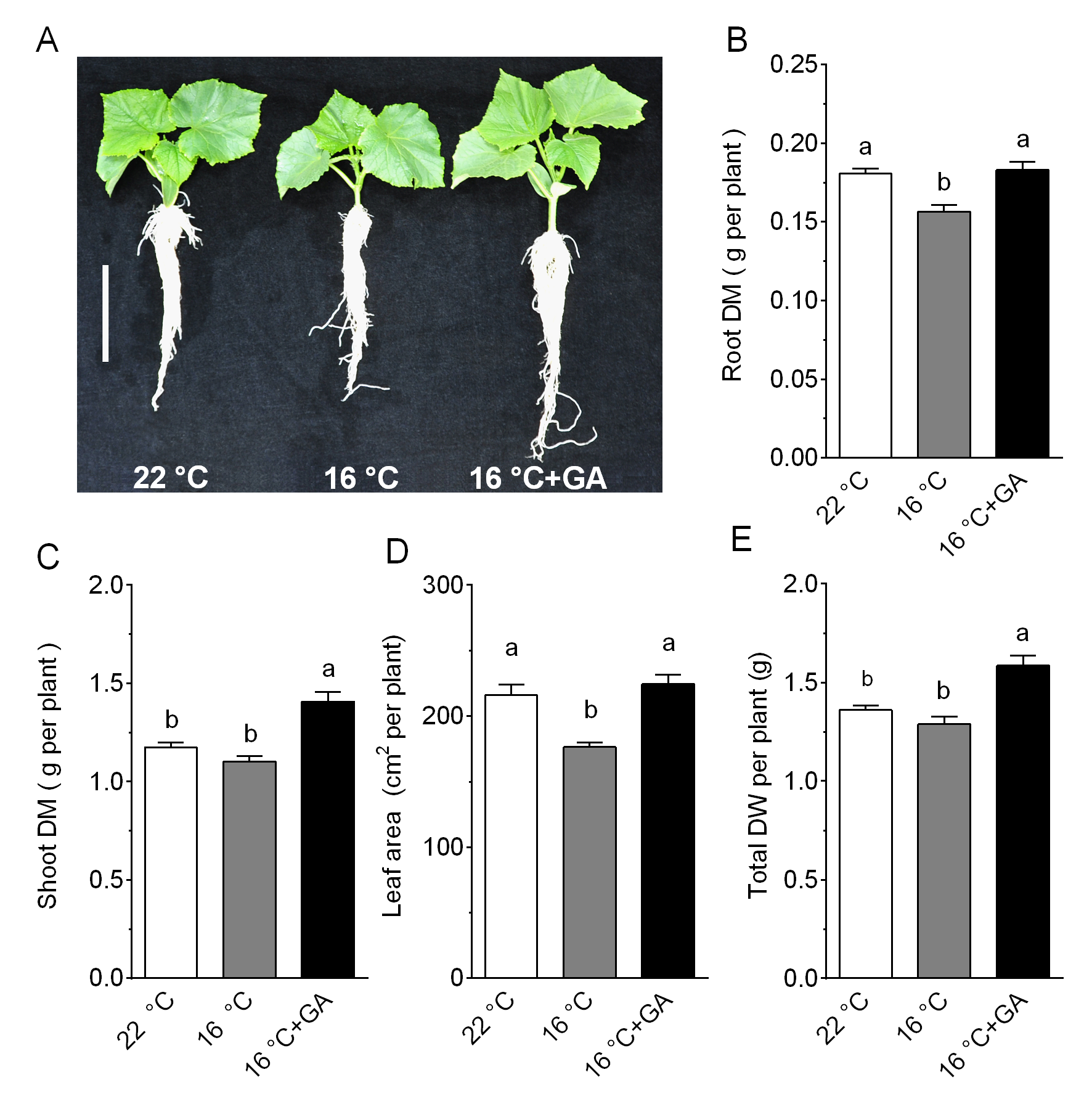

Supplement: S1 Fig — (A) phenotypes of cucumber seedlings. (B) root DM of cucumber seedlings. (C) shoot DM of cucumber seedlings. (D) leaf area of cucumber seedlings. E, root to shoot ratio of cucumber seedlings. 20-day-old cucumber seedlings were transferred to 22 °C Tr and 16 °C Tr conditions in the presence or absence of GA 5 μM GA for 8d. Data are means±SE. Different letters on the top of column indicate significant differences (P <0.05, n = 6). (TIF) [file pone.0156188.s001.tif]
