# Supplementary material for: Gibberellin Is Involved in Inhibition of Cucumber Growth and Nitrogen Uptake at Suboptimal Root-Zone Temperatures
Source: PLoS One. 2016 May 23;11(5):e0156188. doi: 10.1371/journal.pone.0156188 (PMC4877016; doi:10.1371/journal.pone.0156188)
Supplement: S1 Table — (DOCX) [file pone.0156188.s002.docx]

**S1 Table.** The list of gene specific primes for qRT-PCR

| Gene | Primer sequence(5′-3′) | |
| --- | --- | --- |
| *CsGA20ox1* | Forward | CCATTGTGGTTATGCCAGTAGTT |
|  | Reverse | TATCCGAGTTTGGTGCGAAG |
| *CsGA20ox2* | Forward | TAAATCATTGTTCGGTTGCG |
|  | Reverse | GAGAATAGTGAGTGAAGTTGGGTC |
| *CsGA20ox3* | Forward | CCACTTGCTCAACTCGGTCTC |
|  | Reverse | GCTCCTTGTTTCTCCTCCACTT |
| *CsGA20ox4* | Forward | TGGCTACCTTATCCGTTCCTTA |
|  | Reverse | CCTCATTATTGATTCATTGTCCTC |
| *CsGA20ox5* | Forward | TCTCCTCCCACTCCTAACGC |
|  | Reverse | CGGAAGAATGCCAATGACC |
| *CsGA3ox1* | Forward | TGTCCCTCCGACGGTATCT |
|  | Reverse | AACGCAACCCACATCAGC |
| *CsGA3ox2* | Forward | TGAAGGCGAAACGAGAAGAC |
|  | Reverse | ACGGAACGGTTGGTAGTTAGAA |
| *CsGA3ox3* | Forward | GTGCCGAAATTTCACCGCTT |
|  | Reverse | GCGGAAAGGCGGATAGATGA |
| *CsGA3ox4* | Forward | ACACACCGACACCTCCCTCT |
|  | Reverse | AGTAACCCACCCTGTTCCATC |
| *CsGA2ox1* | Forward | GTAGGGTGAACCATTATCCGC |
|  | Reverse | TCCATTCCCATCAGCAAGAG |
| *CsGA2ox2* | Forward | CTTCTTCTTCCCTCCCACGA |
|  | Reverse | CGCATCCTAAGTCTCTCACACC |
| *CsGA2ox3* | Forward | GCTCAACCTCAAAGACCCAG |
|  | Reverse | GCTATTCAATCCAACCCACAT |
| *CsGA2ox4* | Forward | ACCGATAAGGGCTGGATTG |
|  | Reverse | CTCGTCACCGCTGTTACATACT |
| *CsGA2ox5* | Forward | CAAGTGGAAGAGGTGGAACAA |
|  | Reverse | AGTAATAGAAGTGGCGGAGGG |
| *CsNRT1.1* | Forward | TCATTGGTGTCGGTTTAGTTCTGT |
|  | Reverse | TGATAGCCCTGTGCTCATTGTT |
| *CsNRT1.2A* | Forward | CGTGTGCGAAGAAGCGAGT |
|  | Reverse | CAACGACCCCTTTATGCCA |
| *CsNRT1.2B* | Forward | ACATCTCGTTCTCCCAGTTGC |
|  | Reverse | TGAAATCAGCCGACCCTAAA |
| *CsNRT1.3* | Forward | ACTTTTCATCAGAGAAGCACCG |
|  | Reverse | CACACAGCGAGTAGCCAATAGA |
| *CsNRT1.4A* | Forward | CGTTGTCACTTGGGTTCTTTG |
|  | Reverse | GTTTGGGTTTCTGTGGCTTG |
| *CsNRT1.4B* | Forward | CGACACAACTACCCTACCGATA |
|  | Reverse | GCTGAAACCAAGAAACTACTGATG |
| *CsNRT1.5A* | Forward | TGTTTACATTCTCAGTGTCGCAG |
|  | Reverse | TCAGTCGCCTTTAGCATACTTTAG |
| *CsNRT1.5B* | Forward | CAACCAAATCAAGCCTCACAG |
|  | Reverse | CGTTAGTCGGAAAATCTCTACAGT |
| *CsNRT1.8* | Forward | GATGATGACGGAAAGGAAAGC |
|  | Reverse | CAAAGCCAGATTGGGAGCA |
| *CsActin* | Forward | TTCTGGTGATGGTGTGAGTC |
|  | Reverse | GGCAGTGGTGGTGAACATG |
